# Establishment of Pediatric Medication Therapy Management: A Proposed Model

**DOI:** 10.3390/pharmacy4010005

**Published:** 2016-01-19

**Authors:** Sandra Benavides, Shirin Madzhidova, Anihara Hernandez, Thao Le, Stephanie M. Palma, Steffi Stephen

**Affiliations:** 1 Larkin Health Sciences Institute, College of Pharmacy, 18301 N. Miami Avenue, Suite 1, Miami, FL 33169, USA; 2 College of Pharmacy, Nova Southeastern University, Davie, FL 33328, USA; SM93@nova.edu (S.M.); AH984@nova.edu (A.H.); TL644@nova.edu (T.L.); SP931@nova.edu (S.M.P.); SS1820@nova.edu (S.S.)

**Keywords:** pediatrics, medication therapy management, pharmacy, ambulatory, community

## Abstract

Ongoing healthcare reform calls for increased accessibility, enhanced delivery, and improved quality of healthcare. Children and adolescents are experiencing a rise in the prevalence in chronic diseases leading to an increased utilization of medications. The increased use of chronic medications can lead to more medication errors or adverse drug events, particularly in children and adolescents using multiple chronic medications. These ongoing changes expand opportunities for a pharmacist to become further integrated in the inter-professional healthcare delivery for pediatric patients, particularly in an ambulatory or community setting. To date, a systemic process for the provision of medication therapy management (MTM) services in pediatric patients has not been elucidated. The purpose of this paper is to describe a proposed model for delivering pediatric MTM. Furthermore, based on the available literature related to pediatric patients at risk for medication errors, adverse drug reactions, and subsequently-increased utilization of emergency departments and hospitalizations, a set of criteria is proposed for further research investigation.

The passage of the Patient Protection and Affordable Care Act (known as the Affordable Care Act) focuses on the transformation of the delivery of healthcare in terms of quality, efficiency, and accountability [1]. The ACA fosters the exploration of new healthcare delivery models, reimbursement for healthcare delivery focused on collaborative inter-professional integrated care, quantifying and reporting quality of care, and improving care for health conditions that result in frequent hospitalizations [2]. Examples of new healthcare delivery models include medical home/neighborhoods, accountable care organizations, population health management systems, and retail clinics [3]. Healthcare systems are also experiencing a shift from *fee-for-service* to *a pay-for-performance* reimbursement as a result of the law. Examples include Hospital Value-Based Purchasing, Hospital Readmissions Reduction Program, and Medicare Shared Savings Program. Increasingly, reimbursement from Medicare and Medicaid to healthcare providers will also be based on a pay-for-performance model rather than fee-for-service, which will require documentation of improved outcomes in patients.

The focus of healthcare reform provides ample opportunity for a pharmacist to be integrated in the delivery of healthcare for pediatric patients in an effort to improve quality and decrease cost of healthcare, particularly related to medications. Although pharmacists in various settings currently provide care to pediatric patients, limited published data exists on the provision in non-acute care setting, such as an ambulatory or community setting. In the existing published literature on all provisions of pharmacy care in pediatrics (acute and non-acute), no consistent model of service is described. While several processes of direct patient care have been described [4], a systematic and consistent process should be established as the provision of pharmacist provided care expands to pediatric patients in the ambulatory setting. Although the practice in pediatric ambulatory care is expanding, the practice is relatively new and unfamiliar to other health care providers necessitating a focused care process to build relationships, credibility, and value with healthcare providers and patients or caregivers of patients. The most appropriate model to serve as the structure to accomplish a systematic approach of the aforementioned services is through medication therapy management (MTM). Medication therapy management (MTM) is known to other healthcare providers and is an established model with specific requirements in adults defined by the Centers for Medicare and Medicaid Services (CMS). To date, a defined process for delivery of MTM for children has not been described or implemented. The purpose of this manuscript is to propose a systematic process of MTM to be conducted for pediatric patients, and an eligibility criterion that would capture patients that may benefit most from such services.

## 1. Medication Therapy Management

Medication therapy management became a required benefit for all Medicare Part D beneficiaries through the enactment of the Medicare Modernization Act of 2003 (MMA) [5]. The intention of MTM is to improve medication use by optimization of therapeutic outcomes and reducing adverse drug events (ADEs) in high risk patients. In the Medicare population, eligibility for MTM requires (at a minimum) that beneficiaries meet the following criteria: (1) the presence of multiple chronic diseases; (2) take multiple prescription medications; and (3) incur a specified dollar amount for covered Part D drugs. Since pediatric patients are not beneficiaries of Medicare, implementation of MTM for this population has been limited [6,7].

## 2. Necessity of Medication Therapy Management in Pediatric Patients

Although pediatric patients are not current beneficiaries of MTM, the need is becoming increasingly necessary. The rate of chronic diseases or conditions in children is increasing, with the most current estimate at approximately 27% in 2006 [8]. The increase in chronic conditions is attributed to several factors. Improvement in treatment of chronic diseases, such as cystic fibrosis and sickle cell disease, has extended life expectancy. Changes in the environment, such as increased pollution and increased consumption of energy dense foods, have impacted the incidence of asthma and obesity, respectively. Advances in perinatal and neonatal medicine has increased survival of once fatal neonatal conditions but has resulted in chronic conditions such as bronchopulmonary dysplasia. Over the years, there has also been an increased recognition and diagnosis of behavioral disorders, such as attention-deficit hyperactivity disorder (ADHD) and autism. Notably, between 2001 and 2011 the percentage of children with a disability increased by nearly 16% [9]. The percentage of children with a physical disability decreased by 12% in that same time frame, whereas those with a neurodevelopmental or mental health disability increased by 21%, with a largest increase in children less than six years of age [10].

The increase in prevalence of chronic diseases in children leads to an increase use of medications. One study describes an increased utilization of medications for asthma, ADHD, and contraception in children aged 0–17 between the years 2002 and 2010 in the outpatient setting [9]. Another study evaluated the changes in the utilization of chronic medications between 2002 and 2005. The authors reported an increase in the prevalence of medication use by 103% in antidiabetic medications, 63% in ADHD medications, 47% in asthma controller medications, and 15% in antihyperlipidemic medications [11].

It is not uncommon for pediatric patients to also consume complementary and alternative medicines (CAM), such as herbal supplements, vitamins, and minerals in addition to prescribed medicines. The 2007 National Health Interview Survey conducted by the National Institutes of Health, reported the use of CAM among children to be an estimated 12% [12]. In a follow up survey conducted in 2012 on the use of CAM among U.S. children, overall prevalence remained at 11.6%. The most common CAM approach reported in the survey was the use of non-vitamin, non-mineral dietary supplements in which 35.5% of children were using these products to treat a specific health condition [13]. A similar national health survey evaluated predictors of dietary supplement use by children. Of a reported 37% of children that used dietary supplements, 3% reported use due to increased disease burden and healthcare [14]. Although the majority of CAM use in children is controversial and lacks sufficient data to support use, it continues to increase steadily in the US. It is critical to identify and document each therapy used by children to evaluate potential drug interactions and adverse events.

With the increased use of medications, herbals, vitamins, and supplements comes an increase in the potential for a medication error or an ADE. Although the true extent of medication errors in the outpatient setting have not been clearly described, recent studies in general pediatrics and specific chronic diseases in children indicate a high rate of medication errors [15,16,17]. Unfortunately, some of these errors have caused deleterious consequences leading to emergency department visits and hospitalizations. One study evaluated the potential for dosing errors in ambulatory pediatric patients at a health maintenance organization [15]. The authors determined approximately 15% of all prescriptions in the study were over- or under-dosed. The medications most often associated with an error in pediatrics included analgesics (15% over-dosed), antiepileptic medications (35% under-dosed), asthma and allergy medications (12% over-dosed), and psychotrophic medications (5% under-dosed and 7% over-dosed). Specific medications with the highest percentage of dosing errors included oxycodone, azithromycin, phenytoin, valproic acid, hydroxyzine, and methylphenidate. Additionally, the study determined that children had an increased risk for a medication error, particularly when they received more than five prescribed medications (OR 3.3, 95% CI 1.4–7.7) [15].

The rate of ADEs in pediatric patients is estimated to be 15% in a general pediatric practice [16]. Although only 3% were considered to be preventable, 70% of these occurred in the “administration” phase of the medication use process and could have been minimized with communication to a healthcare provider, notably the physician. However, in the study, a parent contacted the prescriber less than 50% of the time when an event occurred. In those who did contact a prescriber, 14% did not do so immediately, resulting in a longer experience of the adverse event. As such, the authors concluded that over 70% of the preventable ADEs could have been avoided through increased communication with the prescriber. Pharmacists can play a role in bridging this gap by discussing what to expect in terms of adverse events and when it is necessary to contact the prescriber.

The potential for medication errors and harm associated with medication errors increases in the pediatric patient with a chronic disease or impairment. A study evaluated the rate of medication errors in pediatric patients with cancer during home administration of chemotherapy [17]. In the study, the investigators conducted medication reviews to detect discrepancies between the prescribed and administered regimens. Additionally, the caregiver for the child was directly observed by a research nurse to determine if errors in the administration of medications occurred. The overall weighted rate of errors in the study was 70.2 per 100 patients (95% CI, 58.9–81.6). Of the total 72 errors in 92 home visits, four resulted in injury and 40 others in potential injury. Most commonly, errors occurred with non-chemotherapy medications and during administration, particularly when caregivers did not increase or decrease doses as directed by the prescriber. Once again, the researchers concluded improved communication could have decreased the number of errors occurring.

Although many medication errors and ADEs in children and adolescents go unnoticed or untreated, medication errors and ADEs can be attributed to 0.5%–3.3% of emergency department visits and 0.16%–4.3% of hospitalizations [18]. Pediatric patients with a complex chronic disease have an increased risk for an ADE related emergency department visit (OR 4.76, 95% CI 4.45–5.10) compared to those without a complex chronic disease. For those patients, the medications most often related to an ADE related emergency department visit include: psychotropics, antimicrobial agents, anticonvulsants, hormones/steroids, and analgesics. One study evaluating Medicaid pediatric beneficiaries described the subset of pediatric patients with highest healthcare utilizations, notably emergency department visits and hospitalizations [19]. Those at increased risk for healthcare utilization used five or more medications for at least 31 days. The authors also identified that all patients on less than four prescribed medications, for an extended period of time (greater than 31 days), also had increased utilization of the emergency department and hospitalizations. The authors of the study concluded high risk pediatric patients may have benefited from a periodic medication review or visit from a pharmacist.

A systematic review conducted in an effort to delineate strategies to decrease hospitalizations for medically-complex children identified potential modifications to the current healthcare system [20]. Two specific strategies that pharmacists can impact, particularly during the process of MTM, include patient’s adherence to prescribed treatments and increased communication across various healthcare settings and disciplines.

Given the increase in chronic conditions and medication use in pediatric patients, the effectiveness of pharmacy-provided care require study utilizing a systematic method. Studies evaluating outcomes secondary to the provision of MTM in the adult population are limited with methodological concerns (e.g., lack of randomization, confounding factors) limiting generalizability [21]. In a meta-analysis evaluating the benefits of MTM in adult patients, only four of the 44 studies meeting inclusion criteria were determined to be of low or minimum risk of bias. Analysis of these studies for various clinical outcomes, such as disease specific biomarkers or readmission rates found minimal benefit to MTM. Some benefits of MTM were noted in patient adherence to medications, medication appropriateness and dosing, health care cost, hospitalization rate (patients with heart failure), hospitalization risk (patients with diabetes), and cost of hospitalization (patients with diabetes). Despite these findings, the benefits were inconclusive and require further, better designed clinical trials. The authors stress the importance of systematic evaluation of MTM programs based on current definitions, taxonomies, and services, of which do not exist at this time in pediatric patients. In light of the lack of definitions, the following is a proposed systematic process to evaluate the impact of MTM in pediatric patients.

## 3. Elements of Medication Therapy Management

Medication therapy management includes five core elements [22]:

(1) Medication therapy review (MTR)
(2) Personal medication record (PMR)
(3) Medication-related action plan (MAP)
(4) Intervention and referral
(5) Documentation and follow-up.

Although many pharmacists are already performing one or more aspects of the required elements in a variety of pediatric healthcare settings, a systematically defined process is necessary to demonstrate improved outcomes and subsequently expand the MTM benefit to children. In the pediatric population, the five core elements should be utilized. However, one hallmark difference between MTM in the pediatric population *versus* the adult is that the parent or caregiver will be more actively involved in the process of providing care to the child.

### 3.1. Medication Therapy Review (MTR)

The purpose of the MTR is to improve the patient’s/caregiver’s understanding of the medications, identify and resolve any ongoing medication related problems, answer questions the patient/caregiver may have about a medication and, most importantly, empower the patient/caregiver to self-manage/manage medications and health conditions. The MTR has three components, (1) collecting information from the patient/caregiver, medical records, or laboratory results when available; (2) assessing the gathered information to detect medication-related problems; and (3) developing a plan for any identified problems [22].

The first step includes gathering all the necessary information. The pharmacist interviews the patient/caregiver to obtain a comprehensive history. The information collected includes basic demographic information, medical history, medication history, immunization status, and social history, when pertinent. Other important information to gather is an assessment of adherence, goals of therapy, cultural issues that may impact medication use, language barriers (if any), literacy level, and education level of the patient/caregiver. Most importantly, the pharmacist should obtain information related to concerns regarding any particular medication or health condition. If available, recent laboratory results, vital signs, or results of a recent physical examination are gathered. If necessary, the pharmacist can conduct a focused physical assessment to ascertain the presence of any adverse reactions to medications. A guide illustrating important factors to consider in pediatric patients during a medication review process has been published [23].

Once the information is gathered, the pharmacist assesses the medication profile for appropriate indications, efficacy, and toxicity (or adverse reactions). The pharmacist describes efficacy related to the goals of therapy as defined by the prescriber, pharmacist, or patient/caregiver (ideally, all three in collaboration). The pharmacist will then develop a list of medication related problems and prioritize the list to determine which problem(s) need to be addressed immediately.

An MTR can be either comprehensive or targeted. In a comprehensive MTR, the pharmacist is completing a global assessment of all medications the patient is taking, whereas the focused MTR may be limited to a particular medication or medication related problem. In adults, a comprehensive MTR is recommended yearly to be performed during the annual well visit, with focused MTRs performed as needed for any new or ongoing medication related problem(s). However, any time a patient is discharged from a hospital, has an emergency department or urgent care visit, a change in a health condition, or a change in financial resources, is an appropriate time for a comprehensive MTR. In pediatric patients, it is appropriate to conduct a comprehensive MTR during an annual well visit. The recommendations for a focused MTR should be similar to the adult recommendations.

### 3.2. Personal Medication Record

The purpose of the PMR is to empower the patient/caregiver to be more involved in the management of their medications (or the child’s medications). The PMR is a comprehensive list of the patient’s medications, including all prescription, over the counter, herbals, vitamins, and supplements. The patient/caregiver is instructed to take the PMR to all encounters in healthcare (e.g., specialist appointments, hospitalizations) to communicate to providers all current medications. Additionally, after each encounter, any changes to medications should be updated in the PMR by the patient, physician, pharmacist, or another healthcare provider.

The PMR should be specific and clear; each prescription should include the specific dose (especially with unit measures when medications are liquid), route of administration, indication, directions of use, specific instructions if needed, and the stop date or expiration date. A PMR should be written at a level understood by the patient/caregiver. For example, in place of “indication”, the use of “take for” is more appropriate. The information that is typically included in the PRM is listed in Table 1.

**Table 1:** Components of a Personal Medication Record.

Patient name
Patient date of birth
Caregiver’s name/relationship (e.g., parent, guardian, nurse)
Caregiver’s phone number
Emergency contact information
Primary care physician
Pharmacist
Pharmacy name and number
Allergies (including food, environmental) with reaction/date
Date last updated (by pharmacist)
Date last reviewed by a healthcare provider
Medications (name, dose, indication, sig, start date, stop date if applicable, prescribing physician)

### 3.3. Medication-Related Action Plan

The purpose of the MAP is to list any actions pertaining to medications the patient/caregiver should complete. The MAP is intended for the patient/caregiver and as such, it only contains actions the patient/caregiver can act upon. For example, if the patient/caregiver is told to begin taking a multi-vitamin, that will be placed on the MAP. As the MAP is intended for the patient, it must, once again, be written in a simple, concise, and appropriate literacy level. The information on a MAP includes the patient’s name, primary care physician’s name, pharmacist’s name and contact information, pharmacy information, and date prepared. Each individual action to be completed by the patient/caregiver should be listed with a place for the patient/caregiver to write what action was completed and when the action was completed. Lastly, the pharmacist can include the next scheduled appointment date and time.

### 3.4. Intervention and/or Referral

The purpose of the MTM process, and particularly the intervention, is to ameliorate any medication related problems, optimize pharmacotherapy outcomes, enhance continuity of care, and engage patients in the medication use process. The pharmacist will collaborate with other healthcare providers to resolve any medication related problems identified throughout the MTM session. In some instances, the pharmacist may take action or change current therapy if agreed upon through a collaborative practice agreement. The pharmacist may also contact the prescriber to alert them of any problems or to recommend a necessary change in therapy. Other interventions can include providing patient education to the patient, providing education on monitoring devices, enhancing medication adherence, and monitoring efficacy and safety of medications. When appropriate, the pharmacist should refer a patient to the primary care provider or emergency department if a problem is severe or urgent.

### 3.5. Documentation and Follow-Up

Any intervention is incomplete until it is documented. Documentation is important to facilitate communication between healthcare providers, for compliance with laws regarding patient medical records, billing and reimbursement (when available), professional liability, and to demonstrate the improvement in clinical outcomes. Ideally, the pharmacist can document in the patient’s official medical record (if working in a healthcare facility). However, if the pharmacist is working in a community setting, it is important for the pharmacist to develop a systematic method of documentation. When developing a plan for the patient, the preferred method is the SOAP (subjective, objective, assessment, and plan) format. The SOAP format is a widely-recognized format and will be understood when included in the patient’s medical record or forwarded to other healthcare providers. Although paper is acceptable, the Center for Medicare and Medicaid Services (CMS) encourages adoption of a standardized health information technology (HIT) for documenting MTM [24]. A thorough resource on documentation for pediatric patients is available [23].

### 3.6. Eligibility Criteria for Pediatric Patients

To date, specific criteria for pediatric MTM have not been defined. The proposed criteria in Table 2 are intended as initial guidelines to further evaluate. The criteria listed are created through a review of the literature aimed at identifying children at highest risk of medication errors or adverse events or with high emergency department utilization or hospitalizations. The criteria are also intended to mimic current MTM criteria in the adult population per CMS.

**Table 2:** Criteria for Medication Therapy Management Eligibility in Pediatric Patients.

Criteria	Requirement
Multiple Chronic Diseases	Two chronic diseases or more
Multiple Medications	Five medications for at least 30 days
	Include patients on
	• Asthma medications
	• Antiepileptic medications
	• Opiates
	• Psychotropic medications
Medication Expenditure Threshold	Incurs an annual cost of $1255 for medications

#### 3.6.1. Multiple Chronic Diseases

The first eligibility criteria defined by CMS requires beneficiaries have multiple chronic diseases. However, in 2010 CMS ruled that three chronic diseases are the most that a healthcare plan may require. With respect to the specific chronic diseases, the healthcare plan may target any chronic disease or beneficiaries with specific chronic disease, but CMS does encourage selecting specific chronic diseases in their patient populations. Also, at least five of nine core adult chronic diseases must be included in the list of eligible chronic diseases.

In pediatric patients, the most common or prevalent chronic diseases have not been established. The National Health Interview Survey has attempted to quantify chronic conditions in children by asking a parent/caregiver whether a child had limitations in activities due to any physical, emotional, or mental conditions [25]. In 2009, the conditions commonly associated with activity limitations included speech problems, asthma, mental retardation, ADHD, learning disability, and other psychiatric/neurological complications. While some of these disease states may not carry a pharmacologic intervention, they are not the most appropriate to include in the list of eligible chronic diseases for pharmacists to focus on. Therefore, until clearer estimates of specific diseases seen in children are realized, the eligibility criteria for MTM in children can include two or more chronic diseases.

#### 3.6.2. Taking Multiple Prescription Medications

The second eligibility criteria for MTM in adults includes that the beneficiary must take multiple Part D medications. The threshold set by CMS is between two and eight medications. Therefore, any healthcare plan must offer MTM to patients on at least eight medications covered by Medicare Part D.

In pediatric patients, not only does the total number of prescribed medications lead to increased risk for emergency department utilization and hospitalizations, the duration of therapy can also increase risk [19]. Therefore, it is important to consider both the amount and duration of polypharmacy in children. Some diseases (e.g., cystic fibrosis) may be life-long and require multiple medications used daily for extended periods of time. Other diseases, such as cancer, may have periods of time in which drug utilization is increased. Studies have found that pediatric patients on at least five medications have an increase in adverse reactions, emergency department visits, and hospitalizations [15,19]. Based on these data, the criteria for multiple medications in children should be, at minimum five medications for at least 30 days.

In adults, the healthcare plan can determine if the number of medications includes all medications or only those for a chronic disease. In pediatric patients, it may be warranted to include specific medications due to the higher level of adverse reactions. Based on the current literature, children and adolescents on opiates for chronic pain control should be automatically eligible for pediatric MTM. Other high risk medications include antiepileptic agents, psychotropic medications, and asthma medications.

#### 3.6.3. Annual Cost Threshold

Lastly, for MTM in the adult population, the criteria set forth is an annual cost threshold exceeded by the beneficiary for prescription drugs covered by Part D. In 2015, the annual threshold was $3138. A recent analysis of healthcare expenditures reported that per capita spending for a person greater than 65 years (those eligible for Medicare Part D) is 2.5 times more than that of a person between the ages of 0–18 years [25]. As a result, a reasonable figure to use as an initial annual cost threshold in pediatrics is $1255. The annual cost threshold may change with more information on the total prescription cost expenditures per child on various healthcare plans (e.g., Medicaid, Children’s Health Insurance Program, private insurance).

## 4. Value-Based System

In the current healthcare climate of improved outcomes and cost containment, a traditional fee-for-service for pharmacist-provided care of MTM services in pediatric patients may not be cost-effective. Currently, Medicaid and other payers do not have an existing benefit for MTM in pediatric patients limiting reimbursement for such services. Additionally, limitations exist in the ability of the pharmacist to bill for service. The establishment of various models of health delivery, such as the Patient Centered Medical Home (PCMH) or an Accountable Care Organization (ACO) may lend to increased incorporation of pharmacy provided services in this population. To date, many PCMHs and ACOs in the adult population have pharmacist provided services in various capacities. One ACO has shown a decrease in health care costs through provision of MTM services [25]. Although metrics for incentivized reimbursement are not directed specifically toward the care of children, currently, the Center for Medicare and Medicaid Services (CMS) are developing quality metrics to determine Medicaid and Child Health Insurance Program reimbursement to hospitals. In 2016, the utilization of a newly developed Child Hospital Consumer Assessment of Healthcare Providers and Systems (HCAHPS) Survey will be evaluated [26]. New metrics for quality of care in pediatrics are also being established and measured, such as the use of atypical antipsychotics in pediatric patients. A qualified pharmacist with appropriate training in pediatrics is able to work towards improved quality and increased reimbursement in a health care system (*i.e.*, PCMH or ACO).

## 5. Conclusions

The practice of pharmacy continues to advance in all specialties. In the pediatric specialty, advances have been made in the area of ambulatory care, but further integration into the delivery of healthcare is necessary. Additionally, minimal reports of established pharmacy services in the community have been published [26]. Based on the available data, we propose that MTM should be offered to children who have two or more chronic diseases, take five or more medications for greater than 30 days, and incur an annual cost of $1255 for medications. Further research and implementation of any systematically-developed processes and criteria can expand the scope of practice for pharmacist in the pediatric setting.
